# Perinatal follow-up of children born after preimplantation genetic diagnosis between 1995 and 2014

**DOI:** 10.1007/s10815-018-1286-2

**Published:** 2018-09-05

**Authors:** Malou Heijligers, Aafke van Montfoort, Madelon Meijer-Hoogeveen, Frank Broekmans, Katelijne Bouman, Irene Homminga, Jos Dreesen, Aimee Paulussen, John Engelen, Edith Coonen, Vyne van der Schoot, Marieke van Deursen-Luijten, Nienke Muntjewerff, Andrea Peeters, Ron van Golde, Mark van der Hoeven, Yvonne Arens, Christine de Die-Smulders

**Affiliations:** 1 Department of Clinical Genetics, Maastricht University Medical Center, Maastricht, The Netherlands; 2 School for Oncology and Developmental Biology, GROW, Maastricht University, Maastricht, The Netherlands; 3 Department of Obstetrics and Gynaecology, Maastricht University Medical Center, Maastricht, The Netherlands; 4 Department of Reproductive Medicine, University Medical Center Utrecht, Utrecht, The Netherlands; 5 Department of Genetics, University Medical Center Groningen, Groningen, The Netherlands; 6 Department of Obstetrics and Gynaecology, University Medical Center Groningen, Groningen, The Netherlands; 7 Department of Clinical Epidemiology and Medical Technology Assessment, Maastricht University Medical Center, Maastricht, The Netherlands; 8 Department of Pediatrics, Maastricht University Medical Center, Maastricht, The Netherlands

**Keywords:** Preimplantation genetic diagnosis, Perinatal outcome, Congenital malformations, Children, Follow-up

## Abstract

**Purpose:**

We aim to evaluate the safety of PGD. We focus on the congenital malformation rate and additionally report on adverse perinatal outcome.

**Methods:**

We collated data from a large group of singletons and multiples born after PGD between 1995 and 2014. Data on congenital malformation rates in live born children and terminated pregnancies, misdiagnosis rate, birth parameters, perinatal mortality, and hospital admissions were prospectively collected by questionnaires.

**Results:**

Four hundred thirty-nine pregnancies in 381 women resulted in 364 live born children. Nine children (2.5%) had major malformations. This percentage is consistent with other PGD cohorts and comparable to the prevalence reported by the European Surveillance of Congenital Anomalies (EUROCAT). We reported one misdiagnosis resulting in a spontaneous abortion of a fetus with an unbalanced chromosome pattern. 20% of the children were born premature (< 37 weeks) and less than 15% had a low birth weight. The incidence of hospital admissions is in line with prematurity and low birth weight rate. One child from a twin, one child from a triplet, and one singleton died at 23, 32, and 37 weeks of gestation respectively.

**Conclusions:**

We found no evidence that PGD treatment increases the risk on congenital malformations or adverse perinatal outcome.

**Trial registration number:**

NCT 2 149485

**Electronic supplementary material:**

The online version of this article (10.1007/s10815-018-1286-2) contains supplementary material, which is available to authorized users.

## Introduction

Preimplantation genetic diagnosis (PGD) offers couples at high risk for transmitting a genetic disease to their offspring an alternative to prenatal diagnosis or may enhance their probability of an ongoing pregnancy if one of the parents is carrier of a structural chromosomal abnormality [1]. Couples opting for PGD have to go through IVF treatment with or without ICSI to obtain embryos for genetic analysis. Blastomeres or trophectoderm (TE) cells for diagnosis are procured through embryo biopsy and analyzed by either polymerase chain reaction (PCR), fluorescence in situ hybridization (FISH), or array-comparative genomic hybridization (array-CGH) in order to determine a specific genetic defect or a chromosomal abnormality [2].

It is hypothesized that assisted reproductive technologies, especially more invasive techniques like ICSI, increase the risk on birth defects [3, 4]. The need for embryo biopsy makes PGD an even more invasive procedure than IVF(-ICSI) treatment alone. It should also be taken into account that the majority of the PGD couples have no history of fertility problems and alternatively could choose for a natural conception with or without invasive prenatal testing. Consequently, the safety of PGD is an issue that needs constant scrutinizing, the more since the number of pregnancies after PGD is increasing over the years [5]. Since the first PGD treatment was performed in the early 1990s [6], clinical studies on PGD pregnancies and children born after PGD did not report a higher rate of major congenital malformations when compared to IVF-ICSI children [7–12]. The results of these studies raise concerns about adverse perinatal and neonatal outcome, like prematurity rate, the incidence of low, and very low birth weight as well as the incidence of perinatal mortality. However, the reported data are derived from only one large and three small cohorts which makes collection of more data desirable. Also, these cohorts mostly contain data on pregnancies and children born after preimplantation genetic screening (PGS), and thus include couples with a history of infertility or advanced maternal age.

This study presents mostly prospectively collected data concerning the perinatal outcome of all PGD pregnancies in the Netherlands between 1995 and 2014. PGS pregnancies are not included. In view of earlier reported concerns and accompanying importance for patients and health care providers, the aim of this study is to establish the safety of PGD in the Netherlands and to contribute to the justification of PGD as a tool in heritable disease prevention. We focus on the congenital malformation rate and additionally report on misdiagnosis, birth parameters, and on perinatal mortality and morbidity.

## Materials and methods

### Study subjects

The study describes results from 439 PGD pregnancies and 366 newborns, originating from all PGD cycles started between 1995 and 2014 in the Netherlands. Two hundred and forty IVF-ICSI treatments were performed in the Maastricht University Medical Centre+ (MUMC+), 161 in the University Medical Centre Utrecht (UMCU), 36 in the University Medical Centre Groningen (UMCG), and two in the Amsterdam Medical Centre (AMC). The latter three IVF departments are PGD transport centers [13]. All genetic analyses were performed at the department of Clinical Genetics of the MUMC+.

### PGD procedure

Ovarian downregulation and follicular growth stimulation protocols were performed according to local protocols, as well as the procedures for IVF or ICSI, but they resemble the procedures applied at the MUMC+ [14, 15]. On day three after oocyte retrieval and fertilization by IVF (only for FISH analysis) or ICSI one or two blastomeres were removed from cleavage stage embryos for genetic analysis. A laser was used to make a hole in the zona pellucida and replaced the use of acidic Tyrode in the early years of PGD. Blastomeres were analyzed using PCR in case of monogenetic or mitochondrial conditions and FISH analysis in case of chromosomal abnormalities. Recently, array-CGH (aCGH) has replaced the FISH technique. Embryos were cultured in individual drops of medium where the development was recorded. On day three or four after oocyte retrieval, one or two unaffected embryos were transferred [16]. For PGD-FISH cases, the genetic analysis only included the chromosomes involved in the translocation. In case of PGD-aCGH, a comprehensive analysis was performed and only normal/balanced-euploid embryos were eligible for transfer. A single embryo transfer (SET) policy has been installed by the Dutch government since 2013, though a preference for SET had already been developed since 2006 [17].

### Study procedure

Data on IVF-ICSI treatments and PGD analyses were collected from the medical files in either the clinical genetics department or the IVF departments of the participating centers. In the first weeks after the birth of their child, the parents filled in a comprehensive questionnaire about the pregnancy and the health of their child. They were asked for consent to gather medical information about pregnancy, delivery, and the health of their child. Additional data regarding pregnancy, delivery, or the newborn were requested from the patient’s gynecologist, the observing midwife practice, or the treating pediatrician, respectively. The collected data included the following: age of both parents at embryo transfer, gravidity, parity, and number of previous IVF/PGD cycles per couple, whether pregnancy was derived from a fresh or frozen/thawed embryo, after usage of IVF or ICSI and PCR, FISH, or array-CGH for analysis, number of blastomeres biopsied, number of transferred embryos, number of live born children, gender, singleton or multiple, PGD indication, major and minor malformations, still births, pregnancy terminations, misdiagnosis, pre- and postnatal genetic testing, term at delivery, birth weight, perinatal mortality, and hospital admissions.

### Statistics

SPSS version 22 (IBM SPSS, Chicago, IL) was used for descriptive statistics. The categorical data are presented as proportions. The continuous data are presented as mean and standard deviation. Further, *z*-scores were calculated for birth weight of all singletons by normalizing the weight of each child using the mean weight with standard deviation of children from a reference population with similar maternal parity, fetal gender, ethnic background, and gestational age (Foundation for Perinatal Registration in the Netherlands 2013).

### Definitions

Congenital malformations are all structural, functional, and genetic anomalies diagnosed in aborted fetuses, at birth, or in the neonatal period. Minor anomalies are those which do not have serious medical, functional, or cosmetic consequences for the child, according to the European Surveillance of Congenital Anomalies [18].

We used the following ICMART definitions [19]: an ongoing pregnancy is defined as having a pregnancy duration of more than 12 weeks. A miscarriage is defined as the spontaneous loss of a pregnancy before 20 completed weeks of gestational age. A still birth is defined as death before the complete expulsion or extraction from its mother, at or after 20 completed weeks of gestational age. Deliveries before 37 completed weeks of gestation are defined premature and a child is born very premature when delivered before 32 weeks of gestational age. Low birth weight is defined as a birth weight less than 2500 g and very low birth weight as less than 1500 g. Perinatal mortality denotes fetal or neonatal death during the second term of the pregnancy (after 20 weeks), during delivery, or until 7 days after the date of birth.

## Results

The characteristics of the couples, treatments, and children are shown in Table 1. Fifty-three percent of the pregnancies originated from the first PGD cycle. ICSI was used for fertilization in 69% of the procedures. The distribution between either one or two biopsied blastomeres was equal. Slightly more often one embryo was transferred.

**Table 1:** Couple, treatment, and child characteristics

Mean maternal age (years + SD)	32.3 ± 3.7
Mean paternal age (years + SD)	35.2 ± 4.5
Number of clinical pregnancies	439
Mode of inheritance in PGD pregnancies	
Autosomal dominant	172/439 (39.1%)
Chromosomal	121/439 (27.6%)
X-linked	77/439 (17.5%)
Autosomal recessive	65/439 (14.8%)
Mitochondrial	4/439 (0.9%)
Nulliparous	245/439 (55.8%)
Cycle order in which pregnancy was achieved	
1	234/439 (53.3%)
2	124/439 (28.2%)
3	74/439 (16.9%)
4	7/439 (1.6%)
Pregnancy after transfer of frozen-thawed embryo(s)	41/439 (9.3%)
Method of fertilization	
IVF	136/439 (31.0%)
ICSI	303/439 (69.0%)
Genetic analysis technique	
PCR	255/439 (58.1%)
FISH	181/439 (41.2%)
Array-CGH	3/439 (0.7%)
Number of blastomeres biopsied per embryo	
1	321/649 (49.5%)
2	328/649 (50.5%)
Number of embryos transferred	
1	241/439 (54.9%)
2	197/439 (44.9%)
3	1/439 (0.2%)
Number of children	364^a^
Boys	165 (45.3%)
Girls	199 (54.7%)
Singleton or multiple	
Singleton	263 (72.3%)
Twin	88 (24.2%)
Triplet	13 (3.6%)

^a^Two children were lost to follow-up

Four hundred and thirty-nine clinical pregnancies resulted in 366 children. Two children were lost to follow-up and excluded from analyses. Thirteen percent of the pregnancies were multiple pregnancies and 28% of the live born children were part of a twin or triplet. In 85.5% of the multiple pregnancies, two embryos were transferred. There was a decline in the double embryos transfer rate and the occurrence of multiple pregnancies over time.

The largest proportion of couples (39%) opted for PGD because of an autosomal dominant (AD) disorder. Huntington disease (12%), myotonic dystrophy (6%), and hereditary breast and ovarian cancer (5%) were the most common AD conditions. PGD for reciprocal translocations (18%) was the most requested indication of all and PGD because of a mitochondrial condition the least (0.9%).

### Safety of PGD

#### Congenital malformation rate

Major malformations were seen in nine of the 364 live born children (2.5%) (Table 2). Four of these children showed multiple congenital anomalies (MCA): two had a chromosomal abnormality (one singleton had trisomy 21 and one of a twin had trisomy 9 mosaicism), one child presented with an atrial septum defect grade II, an orofacial cleft, and a hydronephrosis, and the fourth had an unilateral facial nerve paresis and microtia. The (genetic) cause in the latter two was not found. Three children had a single congenital malformation; a bilateral hydronephrosis, a duodenal web, and a hypospadia, respectively. Isolated congenital heart defects were reported in two children; one atrial and ventricular septum defect and one atrial septum defect in combination with a pulmonary stenosis.

**Table 2:** Congenital malformations, misdiagnosis, and genetic testing in PGD children and pregnancies

Live born	*N* = 364^a^
Major malformations	9 (2.5%)
Multiple malformations	4 (1.1%)
Trisomy 21	1
Mosaic trisomy 9	1 (one of a twin)
ASD, cleft lip, hydronephrosis	1
Unilateral facial nerve paresis, microtia	1
Single malformation	5 (1.4%)
Bilateral hydronephrosis	1
Duodenal web	1 (one of a twin)
Hypospadia	1
Congenital heart disease	2
Minor malformations	5 (1.4%)
Sacral dimple	2
Umbilical hernia	2 (dizygotic twin)
Inguinal hernia	1
Total	14 (3.8%)
Pregnancies	*N* = 439
Still birth	1 (0.2%)
Acardia	1 (one of a triplet)
Pregnancy terminations	3 (0.7%)
Exencephaly	1
Trisomy 18	1
Trisomy 21	1
Genetic testing	*N* (%)
Non-viable fetus/stillbirth	3
8 weeks and 5 days gestational age	1 (trisomy 16)
8 weeks and 6 days gestational age	1 (misdiagnosis)^b^
37 weeks gestational age	1 (balanced karyotype)^c^
Prenatal tests	26/439 (5.9%)
Confirmation PGD diagnosis	9 (all confirmed)
Abnormalities on ultrasound	3 (one trisomy 18, others normal)
Maternal age	6 (all normal)
Increased risk combined test	3 (one trisomy 21, others normal)
Gender testing XL disorder	5 (all girls)
Postnatal tests in the neonatal period	16/364 (4.4%)
Confirmation PGD diagnosis	13 (all confirmed)
Congenital malformations	3 (one mosaicism trisomy 9; one trisomy 21; one normal)

^a^Two children were lost to follow-up and therefore excluded; ^b^[24]; ^c^Intra uterine fetal death

Five children (1.4%) had a minor malformation. Ultrasound of the neonatal spine in two children with a sacral dimple showed closed vertebrae. Three children presented with a congenital herniation: two twin sisters both had an umbilical hernia and a singleton had an inguinal hernia. The twin sisters were born very premature at 29 + 3 weeks gestational age.

An acardiac fetus was detected in a triplet pregnancy and led to a miscarriage. Three pregnancies were terminated because the fetuses were diagnosed with an exencephaly, trisomy 18, and trisomy 21, respectively.

In six pregnancies, ultrasound abnormalities were reported which were not confirmed postnatally. Hydronephrosis was diagnosed four times on ultrasound and one girl was suspected to have an abdominal cyst. In one pregnancy, the three-vessel view was suspect for a cardiac anomaly, but postnatal follow-up showed none (data not further shown).

#### Misdiagnosis

One misdiagnosis is currently known. Chromosomal analysis of a fetus with a negative heart beat on ultrasound showed an unbalanced 47,XX,+der(5)t(X;5)(q13;p14)mat karyotype. The mother was a 46,X,t(X;5)(q13;p14) carrier. PGD analysis was done using FISH (Table 2).

Genetic testing, either, pre- or postnatal, was performed in 10% of the pregnancies. In 53% of the cases, the reason for testing was confirmation of the PGD diagnosis, all were confirmed. Other indications were miscarriages, an increased risk for a trisomic fetus and abnormalities on ultrasound suspect for congenital malformations in the newborn.

#### Birth parameters

Eighty percent of the children were born at term. Eight children (2.2%) were born very premature; all of the latter were from twin pregnancies (Table 3).

**Table 3:** Birth parameters, perinatal mortality, and hospital admissions

	Total	Singletons	Twins	Triplets
Total	364	263 (72.3%)	88 (24.2%)	13 (3.6%)
Term (mean in weeks)	38.6	39.2	36.3	34.1
Premature (< 37 weeks)	63 (17.3%)	20 (7.6%)	35 (39.8%)	8 (61.5%)
Very premature (< 32 weeks)	8 (2.2%)	–	8 (9.1%)	–
Birth weight^a^	*N* = 359	*N* = 262	*N* = 84	*N* = 13
Median in grams	3280	3462	2640	1900
Mean in grams (SD)	3199 (± 699)	3450 (± 533)	2554 (± 610)	2148 (± 696)
Mean *z*-score^b^		0.17		
Low birth weight (< 2500 g)	52 (14.3%)	10 (3.8%)	31 (36.9%)	9 (69.2%)
Very low birth weight (< 1500 g)	9 (2.5%)	1 (0.4%)	8 (9.5%)	–
Perinatal mortality	3 (0.7%)	1 (0.3%)	1 (0.3%)	1 (0.3%)
Hospital admission	67 (18.4%)	36 (9.9%)	25 (6.9%)	8 (2.2%)
Low birth weight	28 (7.7%)			
< 37 weeks	34 (9.3%)			

^a^Data on birth weight were missing of five children

^b^Weight of the individual child minus the mean weight of children of mothers being nulliparous or multiparous as well, who have the same gender and ethnical background and were born at the same gestational age within a reference population, divided by standard deviation (PRN Foundation, 2013). References only available for singletons

*SD*, standard deviation

The mean birth weight was 3450 g (± 533) for singletons with a mean *z*-score of 0.17 (reference: Dutch population of newborn children reported by PRN Foundation, 2013). The birth weight of almost half of the children who were part of a twin was considered to be low or very low, as was the birth weight of more than two third of the children who were part of a triplet. Only one singleton had a very low birth weight. This child weighed 1490 g and was born by selective cesarean section at 35 + 2 weeks gestational age, because of hemolysis, elevated liver enzymes low platelet—syndrome (HELLP) in the mother.

#### Perinatal mortality and morbidity

Perinatal mortality was reported in three pregnancies (0.8%). One boy of a twin died at 23 weeks and 4 days gestational age, following the miscarriage of his sibling at 16 weeks gestational age. One of a triplet showed growth retardation on ultrasound and died in utero at 32 weeks gestational age. A singleton was stillborn at 37 weeks gestational age after an uncomplicated pregnancy. Chromosomal analysis of the latter showed a balanced male karyotype and extensive postmortem examination revealed no dysmorphic features or congenital malformations and no cause for his intrauterine death (Table 3).

Hospital admission was required in 18.4% of the children. The most common reasons for admission were prematurity, (very) low birth weight or a combination of both. Other reasons for admission directly after birth were blood glucose level monitoring, hyperbilirubinemia, and low body temperature. One girl was admitted a week after birth because of a pyelonephritis (Table 3).

Some of the data of the Dutch PGD population are also presented in Supplementary Table 1, as are the data of other PGD cohorts that we have found in the literature and the outcomes of a meta-analysis on IVF-ICSI studies.

## Discussion

PGD is nowadays a worldwide applied technique for the prevention of genetic disease in offspring. Data on the safety of PGD, namely on congenital malformation rate and adverse perinatal outcome, are mostly reported by one specific PGD center or based on data deduced from a small study population. The main objective of this study was to give an overview of data from a large PGD population in order to provide information on the congenital malformation rate, misdiagnosis rate, birth parameters, perinatal mortality, and adverse perinatal outcomes. We report on 364 children and 439 PGD pregnancies. This is data of one of the larger PGD populations published so far.

More girls than boys have been born after PGD treatment, with a ratio of 1.2 for girls. Sex-selection, followed by the transfer of a female embryo, is possible for carrier women of severe X-linked conditions. Corrected for sex-selection in 53 children, a ratio of 1.1 for boys was found, which is in line with the population-based ratio in favor of boys [20].

The major malformation rate in our group of live born PGD children was 2.5%. When including pregnancy terminations due to congenital malformations, this was 3.3%. Other studies of PGD children reported a comparable major malformation rate between 1.7 and 4.1% in live born children (Supplementary Table 1) and conclude this to be equal to the risk on major malformations for IVF-ICSI children. In a meta-analysis, Pandey et al. stated that the relative risk for congenital anomalies in IVF-ICSI pregnancies is 1.67 (1.33–2.09) when compared to natural conceptions and they reported an increase in absolute risk of 2% (1–2%) [4]. However, the European Surveillance of Congenital Anomalies reported a prevalence of 261.45 major and minor birth anomalies per 10,000 births (2.6%) in the period between 2008 and 2012, which is similar to the prevalence in our PGD cohort [18]. Overall, the risk on major malformations in children born after PGD seems not increased when compared to the general population. Our study shows similar results.

In four cases (1.1%) with congenital malformations, a chromosomal abnormality was detected. All of these cases involved autosomal chromosomes and were not related to the PGD indication. Of the two trisomy 21 cases, the mothers that were aged 31 and 34 and the fathers that were 31 and 35 years old were not known to be subfertile and one father did report to be a heavy smoker during the intake interview. The mother of the fetus with trisomy 18 was 34 years old. Influence of paternal age and smoking habits on aneuploidy is still controversial [21]. Liebaers et al. reported on one case with a chromosomal abnormality, in their PGD cohort [12]. Other studies do not report on chromosomal aberrations in their PGD cohort. Bonduelle et al. reported a de-novo chromosomal anomaly rate of 1.6% in ICSI offspring [22]. Results of prenatal diagnostic tests in a large group of naturally conceived women aged 35 years and older showed an incidence of chromosomal abnormalities of 0.9% [23]. Our results seem to be in line with this; however, the mean age of the mothers in our cohort was slightly lower (32.3 ± 3.7). More evidence is needed before any conclusions on the risk of chromosomal abnormalities can be drawn.

The one misdiagnosis described was probably due to a technical error. An unbalanced fetal karyotype is 47,XX,+der(5)t(X;5)(q13;p14)mat. This case has been extensively reviewed [24]. Prenatal genetic testing to confirm the PGD diagnosis was offered to all couples, but was performed in only 10% of the pregnancies. An exact misdiagnosis rate can therefore not be given. Based on our perinatal data, we have no indication to expect more misdiagnosis, bearing in mind that manifestations of late-onset diseases are not yet expected due to the short follow-up time. The European Society of Human Reproduction and Embryology PGD consortium reports a misdiagnosis rate of 0.16%, with the annotation that there is a possibility that not all misdiagnosis have been declared by the PGD centers, and this could be an underestimation [25]. The same consortium evaluated the validity, robustness, and diagnostic value of PCR-based PGD [26, 27]. These data seem reassuring when assessing the safety of PGD.

Almost 20% of the children included in this study were born premature. Less than 15% of the children had a low birth weight. These were mostly multiples. We calculated a *z*-score of + 0.17 for the singletons which indicates a comparable birth weight between our PGD children and the Dutch population. The prematurity rate in the PGD cohorts of Eldar-Geva et al. (15.5%) and Liebaers et al. (28.9%) matches with the incidence of low birth weight in their cohorts, which seems to indicate a correlation [11, 12]. All other studies on PGD and PGS show an evident increase in prematurity in multiples when compared to singletons, as does the risk for low and very low birth weight (see for a summary Supplementary Table 1). Our study distinguished very premature children from premature children which shows that the very premature children were all part of a twin. Bay et al. found an increased risk on adverse birth outcomes in PGD children when compared to children born after naturally conceived pregnancies. [8] The incidence of multiples in their PGD cohort was 30%, compared to an incidence of 3% in naturally conceived children. Considering the possibly higher perinatal risk for multiples this strongly supports the current SET policy. Since gestational age and birth weight in PGD children are comparable to these parameters in IVF-ICSI children it could be suggested that PGD on itself is not an extra risk factor for adverse perinatal outcome [8, 10, 28].

We report one unexplained stillbirth at 37 weeks gestational age. Liebaers et al. reported the perinatal mortality rate to be higher in PGD children (4.64%) compared to ICSI children [12]. However, when they stratified for multiples the increased risk of perinatal death in PGD and ICSI singletons was comparable (1.03%). A meta-analysis by Lamont et al. of cohort and case-control studies describes population-based data on 3,412,079 women from high income countries, of whom 24,541 (0.7%) had a stillbirth [29]. The perinatal mortality rate in our cohort is 0.8%. We found no evidence for a potential increased risk on fetal or neonatal death after PGD.

The incidence of hospital admissions is lower for our PGD children than previously described in PGD cohorts (Supplementary Table 1). These admissions were not associated with the incidence of multiple pregnancies as previously seen in IVF-ICSI cohorts. Explanations for associations found in these latter studies were the higher incidence of preterm birth and low birth weight seen in multiples. There seems to be a relation between the prematurity rate, birth weight, and hospital admissions in our PGD cohort since half of the admitted children were born premature.

This study is one of the first large studies on exclusively PGD pregnancies and children. Other studies reported mostly on children born after preimplantation genetic screening (PGS) and completed their cohort with data on children born after PGD. Contrary to PGD, PGS is offered to couples with an IVF-ICSI indication due to infertility or advanced maternal age and is considered to increase the probability of achieving a pregnancy by excluding aneuploid embryos [30]. Our prospectively gathered data are rather complete as only two children were lost to follow-up. Our results include data on perinatal events and also pregnancy terminations before the perinatal period, loss of children, and congenital malformations in pregnancy. Other studies mostly omitted these latter data. This study population consists of all PGD pregnancies in one country and is not restricted to one specific PGD center or area.

In this report, the data of the Dutch PGD population were compared to published PGD cohorts, mainly from Belgium, and to published data on naturally conceived pregnancies and children and IVF-ICSI pregnancies and children. A future study on the Dutch PGD population with comparison to Dutch IVF-ICSI and naturally conceived children is desirable. Thereby, the children of the Dutch PGD population were not examined by the research group which could introduce an underestimation of the number of, mainly minor, congenital malformations. As Robins et al. reported that over 40% of congenital malformations are diagnosed only after 1 month postpartum [31]. A follow-up study of older PGD children compared to IVF-ICSI children and naturally conceived children is ongoing.

Overall, the risk on major malformations in PGD children seems comparable to children born after IVF-ICSI as to the risk reported in naturally conceived children. Data on pregnancy duration, birth weight, perinatal mortality, and hospital admissions in the Dutch PGD population, especially in the singletons, appear to be similar to the published data on naturally conceived children. In conclusion, PGD does not seem to attribute to an increased risk on an adverse perinatal outcome when compared to naturally conceived children.

## Electronic supplementary material

Supplementary Table 1 (DOCX 23 kb)

## References

[1] de Krom G, Arens YH, Coonen E (2015). Recurrent miscarriage in translocation carriers: no differences in clinical characteristics between couples who accept and couples who decline PGD. Hum Reprod.

[2] Harton G, Braude P, Lashwood A (2011). European Society for Human Reproduction and Embryology (ESHRE) PGD consortium. ESHRE PGD consortium best practice guidelines for organization of a PGD Centre for PGD/preimplantation genetic screening. Hum Reprod.

[3] Davies MJ, Moore VM, Wilson KJ (2012). Reproductive technologies and the risk of birth defects. N Engl J Med.

[4] Pandey S, Shetty A, Hamilton M (2012). Obstetric and perinatal outcomes in singleton pregnancies resulting from IVF/ICSI: a systematic review and meta-analysis. Hum Reprod Update.

[5] De Rycke M, Belva F, Goossens V (2015). ESHRE PGD consortium data collection XIII: cycles from January to December 2010 with pregnancy follow-up to October 2011. Hum Reprod.

[6] Handyside AH, Kontogianni EH, Hardy K (1990). Pregnancies from biopsied human preimplantation embryos sexed by Y-specific DNA amplification. Nature.

[7] Banerjee I, Shevlin M, Taranissi M (2008). Health of children conceived after preimplantation genetic diagnosis: a preliminary outcome study. Reprod BioMed Online.

[8] Bay B, Ingerslev HJ, Lemmen JG (2016). Preimplantation genetic diagnosis: a national multicenter obstetric and neonatal follow-up study. Fertil Steril.

[9] Desmyttere S, Bonduelle M, Nekkebroeck J (2009). Growth and health outcome of 102 2-year-old children conceived after preimplantation genetic diagnosis or screening. Early Hum Dev.

[10] Desmyttere S, De Rycke M, Staessen C (2012). Neonatal follow-up of 995 consecutively born children after embryo biopsy for PGD. Hum Reprod.

[11] Eldar-Geva T, Srebnik N, Altarescu G (2014). Neonatal outcome after preimplantation genetic diagnosis. Fertil Steril.

[12] Liebaers I, Desmyttere S, Verpoest W (2010). Report on a consecutive series of 581 children born after blastomere biopsy for preimplantation genetic diagnosis. Hum Reprod.

[13] Geraedts JPM, Harper J, Braude P (2001). Preimplantation genetic diagnosis (PGD), a collaborative activity of clinical genetic departments and IVF centres. Prenat Diagn.

[14] Dumoulin JC, Meijers CJ, Bras M (1999). Effect of oxygen concentration on human in-vitro fertilization and embryo culture. Hum Reprod.

[15] Dumoulin JC, Land JA, Van Montfoort AP (2010). Effect of in vitro culture of human embryos on birthweight of newborns. Hum Reprod.

[16] Coonen E, Martini E, Dumoulin JC (2000). Preimplantation genetic diagnosis of a reciprocal translocation t(3;11)(q27.3;q24.3) in siblings. Mol Hum Reprod.

[17] van Montfoort AA, Fiddelers AAA, Janssen JM (2006). In unselected patients, elective single-embryo transfer prevents all multiples, but results in significantly lower pregnancy rates compared with double embryo transfer: a randomized controlled trial. Hum Reprod.

[18] European Surveillance of Congenital Anomalies. EUROCAT Guide 1.4 and reference documents. 2013.

[19] Neubourg D, van Duijnhoven NT, Nelen WL (2012). Dutch translation of the ICMART-WHO revised glossary on ART terminology. Gynecol Obstet Investig.

[20] Centraal Bureau voor Statistiek. Geboorte; kerncijfers. 2015.

[21] Templado C, Uroz L, Estop A (2013). New insights on the origin and relevance of aneuploidy in human spermatozoa. Mol Hum Reprod.

[22] Bonduelle M, Van Assche E, Joris H (2002). Prenatal testing in ICSI pregnancies: incidence of chromosomal anomalies in 1586 karyotypes and relation to sperm parameters. Hum Reprod.

[23] Jacobs PA, Browne C, Gregson N (1992). Estimates of the frequency of chromosome abnormalities detectable in unselected newborns using moderate levels of banding. J Med Genet.

[24] van Echten-Arends J, Coonen E, Reuters B (2013). Preimplantation genetic diagnosis for X;autosome translocations: lessons from a case of misdiagnosis. Hum Reprod.

[25] Wilton L, Thornhill A, Traeger-Synodinos J (2009). The causes of misdiagnosis and adverse outcomes in PGD. Hum Reprod.

[26] Dreesen J, Drüsedau M, Smeets H (2008). Validation of preimplantation genetic diagnosis by PCR analysis: genotype comparison of the blastomere and corresponding embryo, implications for clinical practice. Mol Hum Reprod.

[27] Dreesen J, Destouni A, Kourlaba G (2014). Evaluation of PCR-based preimplantation genetic diagnosis applied to monogenic diseases: a collaborative ESHRE PGD consortium study. Eur J Hum Genet.

[28] Sunkara SK, Antonisamy B, Selliah HY (2017). Pre-term birth and low birth weight following preimplantation genetic diagnosis: analysis of 88010 singleton live births following PGD and IVF cycles. Hum Reprod.

[29] Lamont K, Scott NW, Jones GJ (2015). Risk of recurrent stillbirth: systematic review and meta-analysis. BMJ.

[30] Wilton L (2002). Preimplantation genetic diagnosis for aneuploidy screening in early human embryos: a review. Prenat Diagn.

[31] Robins J. Congenital Anomalies Surveillance. Review of data relating to congenital anomalies detected in NHS GG&C between 1^st^ of April 2013 and 31^st^ of March 2014. 2014.

