# Supplementary material for: Perinatal follow-up of children born after preimplantation genetic diagnosis between 1995 and 2014
Source: J Assist Reprod Genet. 2018 Sep 5;35(11):1995–2002. doi: 10.1007/s10815-018-1286-2 (PMC6240547; doi:10.1007/s10815-018-1286-2)
Supplement: Supplementary file 1 — (DOCX 23 kb) [file 10815_2018_1286_MOESM1_ESM.docx]

| **Supplementary Table 1 – Literature overview** | | | | | | | | | |
| --- | --- | --- | --- | --- | --- | --- | --- | --- | --- |
|  | **PGD** | | **PGS and PGD** | | | | | | **IVF/ICSI** |
| **Child characteristics** | **This article** | **Bay (2016)** | **Desmyttere (2012)** | **Liebaers (2010)** | **Desmyttere (2009)** | **Eldar-Geva (2014)** | **Banerjee (2008)** | **Moutou - ESHRE (2014)** | **Pandey (2012)** |
| *Total*   - Singletons (%) - Twins (%) - Triplets (%) | 364  263 (72.3%)  88 (24.2%)  13 (3.6%) | 154  108 (70.1%)  46 (29.9%) | 995  670 (67.3%)  308 (31.0%)  17 (1.71%) | 581  385 (66.3%)  184 (31.7%)  12 (2.07%) | 102  70  32 | 242  158 (65.3%)  84 (34.7%) | 49  41 (83.7%)  8 (16.3%) | 5031  3873  1116  42 | Singletons versus natural conceived |
| Major malformations (%) | 9 (2.5%) | 19 (13.5%) | 23 (2.3%) | 12 (2.13%) | 2 (2.0%) | 4 (1.7%) | 2 (4.1%) |  | N= 4382  RR 1.67 (1.33-2.09); ARI 2% (1-2%) |
| Term (mean in weeks)   - Singletons - Twins - Triplets | 38.6  39.2  36.3  34.1 |  | 38.1 ±2.6  38.7 ±1.9  35.0 ±3.1^a^ | ^d^  38.8 (n=375)  34.9 (n=178)^a^ | 37.7±2.2 | 38.8±1.8  36.6±2.0 | 38.2±2.6 |  |  |
| Premature (<37 weeks)   - Singletons - Multiples | 72 (19.8%)  20 (7.6%)  51 (50.1%) | 29 (20.6%) |  | 160 (28.9%)^d^  43 (11.5%)  117 (65.7%) |  | 37 (15.5%)  15 (9.5%)  22 (26.2%) | 7 (14.3%) | 1361(29.1%)^e^  594 (16.4%)  767 (71.7%) | N = 27819  RR 1.54 (1.47-1.62)  ARI 3% (2-3%) |
| Mean birth weight in grams (SD)   - Singletons - Twins - Triplets | 3199 (±699)^c^  3450 (±533)  2554 (±610)  2148 (±696) |  | 3262.8±543.5  2299.8±581.1^a^ | ^d^  3265±538  2334±566  1290±466 | 2875±658 | 3238±514  2517±475/  2516±454^b^ | 3122±915 | 3222 (n=3385)  2527 (n=1878)  2040 (n=82) |  |
| Low birth weight (<2500 g)   - Singletons - Twins - Triplets | 52 (14.3%)^c^  10 (3.8%)  31 (36.9%)  9 (69.2%) | 26 (18.4%) |  | 138 (25.0%)^d^  28 (7.4%)  110 (62.5%)^a^ |  | 41 (16.9%)  7 (4.4%)  15(36.6%)/  19(45.2%)^b^ | 12 (24.5%) |  | N = 28352  RR 1.65 (1.56-1.75)  ARI 3% (2-5%) |
| Very low birth w. (<1500 g)   - Singletons - Twins | 9 (2.5%)^c^  1 (0.4%)  8 (9.5%) |  |  | 22 (4.0%)^d^  3 (0.8%)  19 (10.8%)^a^ |  | 3 (1.24%)  1 (0.6%)  2 (4.9%)/0^b^ |  |  | N = 27105  RR 1.93 (1.72-2.17)  ARI 1% (1-1%) |
| Perinatal mortality (%)   - Singletons - Twins - Triplets | 3 (0.8%)  1 (0.4%)  1 (1.1%)  1 (7.1%) | 0 (0%) | 36 (3.5%)  8 (1.2%)  26 (8.0%)  2 (11.1%) | 27 (4.64%)^d^  4 (1.03%)  21 (11.41%)  2 (16.60%) |  |  |  |  |  |
| Hospital admission (%) | 67 (18.4%) | 29 (19.5%) |  |  | 35 (34.3%) |  | 8 (16.3%) |  | NICU adm. (N=3530)  RR 1.58 (1.42-1.77) |

^a^ Multiples (twins and triplets); ^b^ First and second twin; ^c^ n=359 children; 262 singletons; 84 twins; 13 triplets; ^d^ 553 children; 375 singletons; 178 multiples; ^e^ 4685 children; 3617 singletons; 1069 multiples

PGD= preimplantation genetic diagnosis; PGS = preimplantation genetic screening; IVF = in-vitro fertilization; ICSI = intracytoplasmic sperm injection; RR = relative risk; ARI = absolute increased risk; SD = standard deviation; gr = grams; w. = weight; adm. = admission
